# E-learning in healthcare professional education: an analysis of political, economic, social, technological, legal and environmental (PESTLE) factors

**DOI:** 10.15694/mep.2019.000097.1

**Published:** 2019-04-25

**Authors:** Kieran Walsh, Lalitha Bhagavatheeswaran, Elisa Roma

**Affiliations:** 1 BMJ

**Keywords:** E-learning, political, economic, social, technological, legal, and environmental

## Abstract

This article was migrated. The article was marked as recommended.

Healthcare professional education is a vitally important part of the healthcare system. E-learning as a means of delivering this education has grown in significance over the years. Research evidence shows that e-learning can help healthcare professionals learn new knowledge and skills. E-learning is fundamentally about education, but it is also a social phenomenon and part of a wider technological revolution. The provision of e-learning for healthcare professional development can be influenced by a number of different factors. These include political, economic, social, technological, legal and environmental factors (PESTLE). All these phenomena influence e-learning in healthcare professional education - their degree of influence often depends on the exact context that is being discussed. A PESTLE analysis uses a framework of these macro-environmental factors that can be used in the strategic analysis of a specific domain. This paper describes an analysis of political, economic, social, technological, legal and environmental factors that can influence e-learning in healthcare professional education.

## The PESTLE approach

### Introduction

Healthcare professional education is a vitally important part of the healthcare system. Excellent education is essential if countries are to continue to improve healthcare for patients and populations. ( Balmer, 2013) E-learning as a means of delivering this education has grown in significance over the years. Research evidence shows that e-learning can be as effective as face-to-face learning and can help healthcare professionals learn new knowledge and skills. ( Cook and Triola, 2014) E-learning is fundamentally about education, but it is also a social phenomenon and part of a wider technological revolution. The provision of e-learning for healthcare professional development can be influenced by a number of different factors. These include political, economic, social, technological, legal and environmental factors (PESTLE). All these phenomena influence e-learning in healthcare professional education - their degree of influence often depends on the exact context that is being discussed. A PESTLE analysis uses a framework of these macro-environmental factors that can be used in the strategic analysis of a specific domain. ( Team, 2013)

It has been used in the analysis of a number of different sectors - including business, organisational, and public sectors. PESTLE analysis enourages thinking that is logical and strategic - rather than thinking that is instinctive or habitual. The above outline explains the general principles of PESTLE. These principles an be applied in any domain. But how might they be applied to e-learning? This paper will outline the political, economic, social, technological, legal and environmental factors that can influence e-learning in healthcare professional education. The purpose is to give a tangible illustration of the application of PESTLE in a healthcare professional education context. The examples described below are not systematic or comprehensive - nor can they be. PESTLE is a tool to be used in “real world” settings and is by its nature pragmatic rather than purist. The examples below are not intended to be the last word on the subject but rather the opening lines of what should be an ongoing discourse.

### Political

Government policies can influence the use of e-learning in healthcare professional education. Governments largely see all forms of healthcare professional education as a means to an end of achieving better healthcare. As such, they will largely be ideologically neutral regarding the issue of what form of education is best and more interested in what will help them deliver the healthcare that the population needs. From a political perspective therefore, e-learning resources should be fully aligned with the political healthcare goals and population needs of the country in question. The e-learning resources should thus be closely mapped to the undergraduate and postgraduate curricula and should be a validated and accredited part of the national continual professional development programme for relevant healthcare professionals. The political climate within a country can also influence the funding landscape for the healthcare sector. Certain subsectors in healthcare, such as medical research, generally attract generous levels of funding and grants. However, in the world of healthcare professional education there is often a dearth of grants. ( Archer *et al*., 2015) Most educational innovations thus rely on the use of existing financial resources for their funding. This can act as a block to radical innovations in the field of e-learning.

### Economic

Until recently little or no attention has been given to the cost of healthcare professional education. However, this has changed and there is now growing interest in the cost and value of education. Most countries want healthcare professional education that is high quality and that can be delivered to sufficient numbers of healthcare professionals at a low cost. In terms of the economics of healthcare professional education, the financial situation in many countries suggests that cost savings are important at present and will continue to be important for the foreseeable future. There are strong reasons to believe that e-learning may be a low cost and high value form of education. ( O’Donovan *et al*., 2016; Walsh and Jaye, 2012) E-learning resources can be produced at a fixed cost and, if simple but effective e-learning resources are created, then this fixed cost can be kept low. ( Walsh, 2015)

The crucial next step is ensuring that e-learning is used and that its usage is scaled up as much as possible. This will ensure the ultimate economic goal in most territories - low cost and high value education. But there is no absolute heterogeneity in economic trends in all territories. For example, in certain economies (especially emerging economies), there is specific and substantial investment in technology and this could have an effect on e-learning in healthcare professional education. Some countries want to develop a thriving technology sector, and this can result in investment in e-learning.

The heterogeneity of different territories means that many are at different stages of market and trade cycles in e-learning. In some countries the economy will be improving and in others it will be deteriorating. In certain countries it may be that investment in the technology sector may be driving an artificial uplift in the investment in e-learning in healthcare professional education.

Market routes and distribution trends can also influence the provision of e-learning in healthcare professional education. Access and availability of resources are important for the dissemination of best practice in healthcare, and e-learning should help with both. However, it is vital that no barriers are placed to accessing the resources - so that maximum possible distribution can be achieved. Barriers may be related to the lack of availability of hardware or software or internet connection to access the resources. Sometimes lack of time can be a barrier to access. ( Kohan *et al*., 2017)

Customer or end-user drivers are other important economic factors in the provision of e-learning resources, as these are the financial buyers of e-learning. The customer of e-learning is often an institution such as a medical school or a postgraduate training authority. Both types of organisation have a requirement to ensure that learners satisfy the needs of educational curricula. E-learning resources can be an effective means of supporting curricula - however it is most important that they are mapped to relevant parts of the curricula. For the end-user, personalisation is often important, and this is increasingly possible with e-learning. E-learning can enable end-users to learn according to their own individual learning needs and to keep a record of their learning and how they have put their learning into action. This can be developed into a substantial electronic portfolio. ( Frank and Gifford, 2017)

### Social

A wide range of social factors can influence the use of e-learning in healthcare professional education. Social factors can include lifestyle trends, population demographics, and major healthcare events. In the past many healthcare professionals were used to travelling to attend educational events. But one recent lifestyle trend is that many people do not want to travel to educational meetings as much as they did in the past. This may be due to various reasons. Some may not find educational events cost-effective and some may not wish to spend time away from their family. Some people may be concerned about the adverse environmental effects of travel. This point will be covered later in the paper. The social demographics of users can also have an effect. It is axiomatic to state that younger people are higher users of technology than older people. At the same time, younger people are more likely to have considerably higher expectations of digital products than their older counterparts. Too often in the past e-learning has just reproduced traditional resources and placed them online - this practice is unlikely to produce resources that will be fit for the future. If e-learning providers are not to disappoint younger users, then they will need to be of a quality that young people will expect. This may mean high levels of interactivity and multimedia within the e-learning resources - all of which are designed to engage the learner and to help them to put their learning into practice. ( Choi-Lundberg *et al*., 2016; Walsh *et al*., 2010) Increasingly learners see themselves as consumers, and this perspective can have an effect on their attitudes and opinions. It is likely that such attitudes will mean higher expectations of digital products - especially if learners are investing their own financial resources to access the e-learning. Indeed, the social consumer buying patterns in healthcare professional education are likely to be changing as a result of e-learning. In the early years of the internet it was assumed that all content on the internet would be free. However, in recent years it has become clear that individuals and their institutions are willing to pay for high quality e-learning resources that meet their needs. This may be driven by the concerns over free e-learning resources. One concern is that free e-learning resources may be sponsored and that the sponsor may have had influence over the content. This would be a threat to the editorial independence and the quality of the content. Another concern around free e-learning resources is around data collection. Sometimes resources are made freely available so that their creators will have access to data on their usage. This data may be valuable to certain stakeholders - but learners may be uncomfortable with their data being used for commercial purposes. The social effects of e-learning can be associated with other ethical issues. These may not be related to the content, but rather to the amount of time that individuals might spend online - with sometimes adverse resultant effects on their mental and physical health. This can be a particular concern for younger people. It is unlikely to be good practice for healthcare professionals to do all their learning online - it is likely to be much more useful for them to blend online learning with face-to-face learning experiences. Major healthcare or social events can also have an influence on the take-up of e-learning. For example, an outbreak of a serious infectious disease (such as an influenza pandemic) can stimulate users to do more e-learning so that they are continually updated with regard to rapidly changing guidelines (which inevitably occurs in the midst of a pandemic). ( Walsh *et al*., 2018)

### Technological

Technological factors naturally play a vital role in the field of e-learning in healthcare professional education. Information technology is continuing to develop rapidly both inside and outside of healthcare professional education and education more widely. This trend inevitably has a significant effect on the role of e-learning. Associated or dependent technologies are required for e-learning to function - examples include the need for hardware, software, and broadband internet connections or Wi-Fi. Fortunately, these technologies are increasingly available around the globe, and so enable the ubiquitous take-up of e-learning. In terms of the maturity of technology, there is little doubt that the technology is still immature; many innovations are still being created and indeed the future innovation potential remains high. As a technology, e-learning has tremendous potential for building capacity and this can enable economies of scale in healthcare professional education. There can be intellectual property issues in terms of both content and technology in e-learning - but their effect largely depends on the territory that is under question. In most Western countries, issues with intellectual property are usually manageable - but they can be a major problem elsewhere. Technology access and licensing can also influence e-learning in healthcare professional education. Some technology platforms are open source - which should further accelerate the provision and take-up of e-learning. In the modern world, technology is increasingly becoming global - yet how the resources are used in different countries has important implications for providers of e-learning. For example, in African countries, mobile technology is becoming ubiquitous. Global providers of e-learning thus need to ensure that their resources will work on a variety of different types of devices (including mobile ones). ( Masters *et al*., 2016) Technology-enhanced learning is a form of communications technology and e-learning can enable personalised messages. These personalised messages can enable providers of e-learning to provide learning resources that are based on learners’ needs. These needs can then be satisfied by making explicit recommendations of content that is based on the needs. A final technological factor that may need to be taken into account is interoperability between different e-learning systems. At present there is little interoperability between different systems, and this is viewed negatively by many learners. It is likely that improved interoperability between different systems would be appreciated a great deal by learners. This is simply so that they can store all their learning in one place.

### Legal

Legal factors can also influence the uptake of e-learning for healthcare professional development. National and international legislation may need to be considered. Certain countries (such as those with repressive regimes) may block access to certain websites. This can effectively block e-learning within that country. On the other hand, in other regions, data protection laws can prevent providers of e-learning from retaining too much data on their learners. Both dynamics need to be considered. Regulatory bodies and processes can also have an effect. Regulatory bodies are increasingly recognising e-learning as a form of continuous professional development. In the early years of e-learning, it was clear that regulatory bodies that are responsible for the accreditation of continuous professional development had been set up to recognise traditional forms of education (such as meetings or conferences). When e-learning started to emerge, they were initially unready to accredit e-learning. However, in the past few years, regulatory bodies have started to develop processes whereby they can accredit e-learning resources or e-learning providers. ( https://www.uems.eu/__data/assets/pdf_file/0017/40157/EACCME-2.0-CRITERIA-FOR-THE-ACCREDITATION-OF-ELM-Version-6-07-09-16.pdf)

### Environmental

Ecological and environmental issues are important influencers in the take up of e-learning in healthcare professional education in many countries. Concerns about climate change and carbon footprint due to travel for continuous professional development will drive the use of e-learning for healthcare professional education. ( Walsh, 2018) Similar concerns will also make print-based resources less popular.

### Conclusions

At its core, e-learning is about education, but it cannot be viewed in isolation from the social, technological, and political context in which it is based. The PESTLE analysis outlined above explores a number of factors that should be considered when setting up e-learning. It is important to consider these factors when thinking of introducing e-learning to a new country or region.

## Take Home Messages

• E-learning as a means of delivering healthcare professional education has grown in significance over the years.
• E-learning is fundamentally about education, but it is also a social phenomenon and part of a wider technological revolution.
• Political, economic, social, technological, legal and environmental factors can influence the provision of e-learning for healthcare professional development - their degree of influence often depends on the exact context that is being discussed.

## Notes On Contributors

Kieran Walsh is Clinical Director at BMJ.

Lalitha Bhagavatheeswaran is Clinical Outreach and Engagement Manager at BMJ.

Elisa Roma is Programmes and Partnerships Manager at BMJ.

## Declarations

The author has declared the conflicts of interest below.

Kieran Walsh works for BMJ, which produces an e-learning resource - BMJ Learning. Lalitha Bhagavatheeswaran works for BMJ, which produces an e-learning resource - BMJ Learning. Elisa Roma works for BMJ, which produces an e-learning resource - BMJ Learning.

## Ethics Statement

This is not original research.

## External Funding

This article has not had any External Funding

## References

[ref1] Archer J. McManus C. Woolf K. Monrouxe L. Illing J. (2015) Without proper research funding, how can medical education be evidence based? BMJ. 350, p.3445. 10.1136/bmj.h3445 26116693

[ref2] Balmer J.T. (2013) The transformation of continuing medical education (CME) in the United States. Adv Med Educ Pract. 4, pp.171–182. 10.2147/amep.s35087 24101887 PMC3791543

[ref3] Choi-Lundberg D.L. Cuellar W.A. Williams A.M. (2016) Online dissection audio-visual resources for human anatomy: Undergraduate medical students’ usage and learning outcomes. Anat Sci Educ. 9(6), pp.545–554. 10.1002/ase.1607 27802370

[ref4] Cook D.A. Triola M.M. (2014) What is the role of e-learning? Looking past the hype’, Med Educ. 48, pp.930–937. 10.1111/medu.12484 25113119

[ref5] Frank A. Gifford K. (2017) Electronic portfolio use in pediatric residency and perceived efficacy as a tool for teaching lifelong learning. BMC Med Educ. 17(1), p.202. 10.1186/s12909-017-1045-6 29126405 PMC5681817

[ref6] UEMS (2016). EACCME CRITERIA FOR THE ACCREDITATION OF ELEARNING MATERIALS (ELM). Available at: https://www.uems.eu/__data/assets/pdf_file/0017/40157/EACCME-2.0-CRITERIA-FOR-THE-ACCREDITATION-OF-ELM-Version-6-07-09-16.pdf( Accessed: 2.4.2019)

[ref7] Kohan N. Soltani Arabshahi K. Mojtahedzadeh R. Abbaszadeh A. Rakhshani T. (2017) Self- directed learning barriers in a virtual environment: a qualitative study. J Adv Med Educ Prof. 5(3), pp.116–123. 28761885 PMC5522903

[ref8] Masters K. Ellaway R.H. Topps D. Archibald D. Hogue R.J. (2016) Mobile technologies in medical education: AMEE Guide No.105. Med Teach. 38(6), pp.537–549. 10.3109/0142159x.2016.1141190 27010681

[ref9] O’Donovan J. Ahn R. Nelson B.D. Kagan C. Burke T.F. (2016) Using low-cost Android tablets and instructional videos to teach clinical skills to medical students in Kenya: a prospective study. JRSM Open. 7(8),2054270416645044. 10.1177/2054270416645044 27540487 PMC4973399

[ref10] Team F.M. (2013) PESTLE Analysis. Strategy Skills. Free management ebooks.

[ref11] Walsh K. (2015) E-learning: controlling costs and increasing value. J Coll Physicians Surg Pak. 25(4), pp.292–293. 25899197

[ref12] Walsh K (2018). E-learning in medical education: the potential environmental impact. Educ Prim Care. 29(2), pp.104–106. 10.1080/14739879.2017.1389619 29050529

[ref13] Walsh K. Jaye P (2012). The relationship between fidelity and cost in simulation. Med Educ. 46(12), p.1226. 10.1111/j.1365-2923.2012.04352.x 23171265

[ref14] Walsh K. Sandars J. Kapoor S. Siddiqi K (2010). Getting NICE guidelines into practice: can e-learning help? Clinical Governance: An International Journal. 159(1), pp.6–11. 10.1108/14777271011017329

[ref15] Walsh K. Sandars J. Nordquist J (2018). Technology-enhanced learning for healthcare professionals: an essential response to infectious disease pandemics. BMJ Simulation and Technology Enhanced Learning. 4, pp.1–3. 10.1136/bmjstel-2017-000236 PMC8936737 35517379

