# Supplementary material for: E-learning in healthcare professional education: an analysis of political, economic, social, technological, legal and environmental (PESTLE) factors
Source: MedEdPublish (2016). 2019 Apr 25;8:97. [Version 1] doi: 10.15694/mep.2019.000097.1 (PMC10712588; doi:10.15694/mep.2019.000097.1)
Supplement: Peer review history

# Reviewer report: Reviewer response for version 1

**DOI:** 10.21956/mep.19836.r29907

**Published:** 2019-06-21

**Authors:** Hassaan Waqar

**Affiliations:** 1 St Helens and Knowsley Teaching Hospitals NHS Trust – Lead Employer

This review has been migrated. The reviewer awarded 3 stars out of 5 This is an exciting area of research within the field of medical education and the authors should be commended for choosing to analyse such an area. The abstract is a concise summary of the themes raised within the article and neatly summaries the main points that are discussed in the PESTLE analysis. The limitations of e-learning are explored, however there is limited analysis of the legal and environmental factors which influence the delivery of e-learning.

Reviewer Expertise:

NA

No decision status is available

# Reviewer report: Reviewer response for version 1

**DOI:** 10.21956/mep.19836.r29908

**Published:** 2019-05-10

**Authors:** Lee HangFu

**Affiliations:** 1 Windsor University School of Medicine

This review has been migrated. The reviewer awarded 3 stars out of 5 This paper provided some important factors that should be considered in the development and advancement of e-learning. The authors have discussed superficially on PESTLE factors. A more in-depth look at the accreditation requirements, cultural "affects" in e-learning, and various personal barriers to e-learning vs. face to face learning need to be examined as the e-learning wave is definitely coming.

Reviewer Expertise:

NA

No decision status is available

# Reviewer report: Reviewer response for version 1

**DOI:** 10.21956/mep.19836.r29906

**Published:** 2019-05-03

**Authors:** Hui Meng Er

**Affiliations:** 1 International Medical University

This review has been migrated. The reviewer awarded 4 stars out of 5 E-learning was introduced more than a decade ago and is evolving rapidly in the recent years due to technological advancement and Industrial Revolution 4.0. However, there is still much to be done to optimise its implementation and penetration, especially in less developed regions. The authors have done a comprehensive analysis in consideration of the PESTLE factors, which are related to the state of readiness for E-learning implementation. They have highlighted relevant issues such as data privacy, ethics and intellectual property concerns that need to be sorted out during the planning phase in order to ensure smooth delivery. Suggestions are made for better interoperability between systems in order to enhance the learners’ experience, as well as the need to work hand-in-hand with the regulatory bodies for accreditation as E-learning is gaining popularity as the mainstream mode of learning. Besides being a tool for educating healthcare professionals, E-learning is also applicable to public education. It would be useful to include some discussion on this aspect, since healthcare professionals will increasingly be encountering patients who engage in health information seeking via internet.

Reviewer Expertise:

NA

No decision status is available
